# Radical Prostatectomy without Prior Biopsy in Patients with High Suspicion of Prostate Cancer Based on Multiparametric Magnetic Resonance Imaging and Prostate-Specific Membrane Antigen Positron Emission Tomography: A Prospective Cohort Study

**DOI:** 10.3390/cancers15041266

**Published:** 2023-02-16

**Authors:** Michael Chaloupka, Maria Apfelbeck, Nikolaos Pyrgidis, Julian Marcon, Philipp Weinhold, Christian G. Stief

**Affiliations:** Department of Urology, LMU Klinikum, Ludwigs-Maximilians University Munich, 80539 Munich, Germany

**Keywords:** radical prostatectomy, mpMRI, PSMA PET, prostate biopsy

## Abstract

**Simple Summary:**

Today, modern imaging techniques can predict advanced prostate cancer with a very high level of certainty. We conducted a prospective observational study of patients undergoing radical prostatectomy for prostate cancer without a prior biopsy. We saw that all patients with elevated PSA levels, a suspicious digital rectal examination, and a high likelihood of prostate cancer on preoperative mpMRI and PSMA-PET imaging were diagnosed with clinically significant prostate cancer. In fact, applying the inclusion criteria of our study, most patients were diagnosed with highly aggressive and locally advanced prostate cancer. In conclusion, we show that in highly selected cases, surgery for prostate cancer is an option.

**Abstract:**

Modern risk stratification of prostate cancer (PCa) allows for prediction of advanced disease with a high level of certainty. We aimed to evaluate a prospective series of patients undergoing radical prostatectomy without prior biopsy based solely on clinical criteria and imaging results. The patients were divided into three groups. Group 1 included 27 patients with: (i) suspicious digital rectal examination, (ii) PSA ≥ 10 ng/mL, (iii) PI-RADS 4/5 on mpMRI, and (iv) high suspicion of PCa on PSMA-PET. Group 2 included six patients who fulfilled criteria i, ii, and iii but did not undergo PSMA-PET imaging. Group 3 included 17 patients with at least one clinical (i or ii) and one imaging (iii or iv) criterion. All of the patients were diagnosed with PCa. Comparison of Group 1 and 2 versus Group 3 showed a significantly higher ratio of locally advanced PCa for Groups 1 and 2 compared to Group 3 (60.6% versus 11.8%, *p* = 0.005, respectively). Similarly, these patients displayed a significantly higher ratio of aggressive PCa (ISUP grade > 2: 66.7% versus 23.5%, *p* = 0.027, respectively) and tumor infiltration (median tumor infiltration: 32.5% vs. 15%, *p* = 0.001, respectively) in the final specimen compared to Group 3. In conclusion, we have shown that radical prostatectomy without prior biopsy is safe in terms of the diagnosis of clinically significant PCa when proper preoperative risk stratification involving mpMRI and PSMA-PET imaging is applied.

## 1. Introduction

Prostate-specific membrane antigen-positron emission tomography (PSMA-PET) imaging and multiparametric magnetic resonance imaging (mpMRI) of the prostate have improved diagnostic workup in prostate cancer (PCa), especially in patients with advanced disease [1,2]. The Australian PRIMARY trial evaluated the use of PSMA-PET/CT alongside mpMRI in biopsy-naïve men with suspicion of PCa undergoing subsequent biopsy and found a sensitivity of 97% for the detection of clinically significant PCa [3]. With the current stage migration of PCa towards locally more advanced disease [4], this raises the question as to whether a biopsy is necessary to confirm cancer.

Prostate biopsy either by a transperineal or transrectal approach is associated with significant morbidity and mortality. Severe complications may occur in up to 2% of patients and include urinary retention, hematuria, rectal bleeding, or sepsis [5]. Accordingly, prostate biopsy defers the time from diagnosis to definite treatment. This often constitutes a huge psychological burden to patients, leading to further preoperative anxiety. It also raises the question as to whether surgical performance is impacted by a prostate biopsy. Retrospective studies suggest an impairment of nerve-sparing during radical prostatectomy [6]. Recently, a small, retrospective case series indicated that in patients with high suspicion of PCa on mpMRI and PSMA-PET, avoidance of prostate biopsy prior to radical prostatectomy may be a valid approach in well-selected and well-counseled patients [7]. To add to these prior findings, we report, to our knowledge, the first ethically approved, prospective cohort study in patients undergoing radical prostatectomy without prior prostate biopsy and based solely on high clinical and imaging suspicion for PCa.

## 2. Materials and Methods

### 2.1. Study Design and Selection Criteria

We performed a prospective, observational study between March 2019 and August 2022 at the Department of Urology, Ludwig Maximilian University of Munich, Munich, Germany and report its findings based on the Strengthening the Reporting of Observational Studies in Epidemiology (STROBE) statement for cohort studies [8]. The present study was approved by the ethics committee (#19-693, #20-1022) of the university and all patients gave written informed consent upon inclusion. Upon referral to our department, all of the patients underwent an extensive discussion with their surgeon about the recommended diagnostic pathway, which includes the necessity to perform a prostate biopsy before proceeding to radical prostatectomy according to current guidelines [9]. Patients who explicitly wished to avoid prostate biopsy prior to radical prostatectomy were included in the present study and were divided into three groups.

Group 1 encompassed patients with: (i) suspicious digital rectal examination (DRE), (ii) PSA ≥ 10 ng/mL, (iii) high suspicion of PCa on mpMRI (PI-RADS 4 or 5), and (iv) high suspicion of PCa on PSMA-PET imaging (CT or MRI) represented by local uptake of PSMA-ligand. The likelihood of PCa in PSMA-PET imaging was not assessed according to a scoring system, but in a dichotomous manner. After evaluation of the preliminary results showing locally advanced or high-risk PCa in all cases, the internal review board of our department granted approval to exclude PSMA-PET imaging as mandatory preoperative imaging in patients who opted for immediate radical prostatectomy. Therefore, Group 2 included patients with: (i) suspicious DRE, (ii) PSA ≥ 10 ng/mL, and (iii) high suspicion of PCa on mpMRI (PI-RADS 4 or 5). Despite the promising preliminary results of Group 1, approval from our internal review board was not granted to perform radical prostatectomy without prior prostate biopsy in patients with at least one clinical criterion (suspicious DRE or PSA ≥ 10 ng/mL) and one imaging criterion (PI-RADS 4/5 on mpMRI or local uptake of PSMA-ligand on PSMA-PET) who could not be classified into Group 1 or 2. Nevertheless, an individual concept was performed in some well-selected patients, who explicitly wished for immediate radical prostatectomy with one clinical and one imaging criterion, despite thorough discussion of possible risks (Group 3).

### 2.2. Study Protocol and Data Collection

All of the patients received open, retropubic radical prostatectomy by a single surgeon with subsequent extended lymph node dissection in cases of suspected lymph node metastasis on mpMRI and/or PSMA-PET imaging. Prior imaging had been performed both in our departments of radiology and nuclear medicine and in external departments, including university and private institutions. In cases of external imaging, mpMRI and PSMA-PET imaging was reviewed by radiologists from our clinic specialized in urologic imaging. For all of the patients, comprehensive demographic data, perioperative clinical parameters, and histopathological information of prostate specimens were available.

### 2.3. Outcomes and Statistical Analysis

The primary outcome of our study was to assess the diagnostic accuracy for clinically significant PCa defined as International Society of Urological Pathology (ISUP) grade >1 after radical prostatectomy based solely on clinical and imaging criteria. Secondary outcomes included: (i) clinicopathological characteristics of the study cohort; (ii) perioperative characteristics of the study cohort; and (iii) diagnostic accuracy of mpMRI and PSMA-PET imaging to predict lymph node metastases without biopsy-proven PCa. All of the continuous variables were summarized as median with interquartile range (IQR) and all of the categorical variables were summarized as absolute numbers with proportions. For all of the analyses, Group 1 and Group 2 were merged and compared to Group 3. For all of the categorical variables, the Fisher’s exact test was applied, and for all of the continuous variables, the Mann–Whitney *U* test was applied. All of the statistical analyses were performed using Prism 6 software (GraphPad Software, San Diego, CA, USA) and two-sided *p*-values ≤ 0.05 were considered statistically significant.

## 3. Results

### 3.1. Clinicopathological Characteristics

The clinicopathological and perioperative characteristics of the study cohort, as well as the between-Group comparisons, are displayed in Table 1.

**Figure 1:**
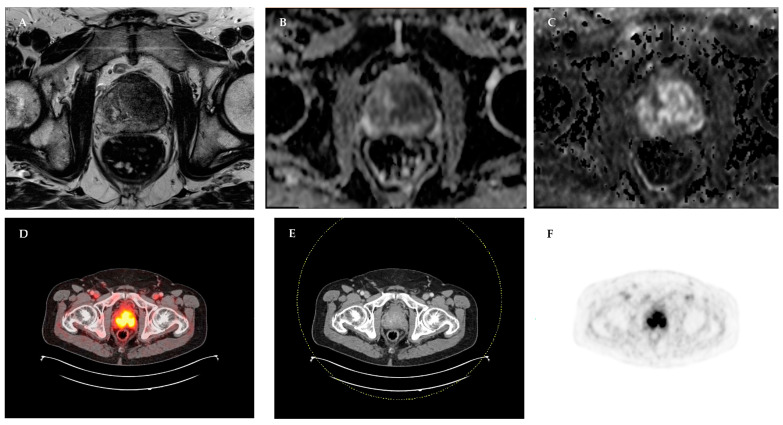
Preoperative mpMRI of the prostate and PSMA-PET/CT scan of a 70-year-old patient with PSA 56 ng/ml and positive DRE (Group 1) shows a large lesion from the apex to the base of the left prostatic lobe with consecutive diffusion restriction (PI-RADS 5) and enhanced uptake of PSMA-ligand. The patient underwent radical prostatectomy without prior biopsy and was diagnosed with prostate cancer ISUP 2 pT3b pN0 R1. (**A**) Axial T2-weighted sequence of mpMRI of the prostate; (**B**) ADC-map of mpMRI of the prostate; (**C**) DWI-sequence of mpMRI of the prostate; (**D**) Fused imaging of PSMA-PET/CT; (**E**) CT scan of the abdomen; (**F**) PSMA-PET scan of the abdomen. *mpMRI*: multiparametric magnetic resonance imaging; *PSMA-PET/CT*: prostate-specific membrane antigen positron emission tomography/computer tomography; *PSA*: prostate-specific antigen; *DRE*: digital rectal examination; *PI-RADS*: Prostate Imaging Reporting and Data System; *ISUP*: International Society of Urologic Pathologists; *ADC*: apparent diffusion coefficient; *DWI*: diffusion-weighted imaging.

An exemplary image of a patient in Group 1 is displayed in Figure 1.

### 3.2. Comparison between the Groups

Patients of Group 1 and Group 2 showed a significantly higher ratio of locally advanced PCa compared to Group 3 (pT3a or pT3b: 60.6% vs. 11.8%, *p* = 0.005). Similarly, these patients displayed a significantly higher ratio of aggressive PCa compared to Group 3 (ISUP grade > 2: 66.7% vs. 23.5%, *p* = 0.027). Accordingly, patients of Group 1 and 2 were diagnosed with a significantly higher ratio of tumor infiltration in the final radical prostatectomy specimen compared to Group 3 (median tumor infiltration: 32.5% vs. 15%, *p* = 0.001). Radical prostatectomies in patients of Group 1 and 2 showed a longer operative time (*p* < 0.001) and higher postoperative hemoglobin decrease (*p* = 0.03) compared to Group 3.

## 4. Discussion

mpMRI and PSMA-PET imaging complemented preclinical risk stratification to a point that PCa could be predicted with high accuracy. We therefore aimed to investigate the feasibility and outcome of performing radical prostatectomy based solely on preclinical risk stratification including mpMRI and PSMA-PET imaging in well-selected and well-informed patients. We showed that by applying the strict inclusion criteria of the study involving mpMRI and PSMA-PET imaging, every patient was diagnosed with clinically significant PCa. This led to significantly more frequent diagnosis of aggressive and locally advanced PCa compared to the retrospective control group.

Surgery without prior biopsy is common clinical practice in other urological and non-urological tumor entities. Current guidelines suggest that biopsy is not mandatory in a contrast-enhancing renal mass prior to planned surgery due to the high diagnostic accuracy of abdominal imaging [10]. Moreover, pancreaticoduodenectomy (Whipple procedure) is routinely performed based on preoperative morphologic imaging such as sonography, CT, MRI, and magnetic resonance cholangiopancreatography [11]. Guidelines recommend to not perform prior biopsy unless it has a significant impact on further treatment and they recommend primary resection of the tumor [11]. However, up to 18% of patients are diagnosed with benign disease after surgery [12]. Preoperative risk stratification prior to Whipple surgery therefore routinely misdiagnoses a large number of patients, unnecessarily impairing health-related quality of life (HRQOL).

Compared to morphology-based imaging before Whipple surgery, PSMA-PET imaging is a PCa-specific modality that can detect PCa even in morphologically unremarkable lesions. In the proPSMA trial, Hofman et al. evaluated the utility of PSMA-PET imaging against conventional morphologic imaging of PCa by CT scan and bone scintigraphy [1]. In this prospective, randomized, multicentric phase 3 trial the authors concluded that PSMA-PET imaging had a 27% higher accuracy in the detection of PCa lesions compared to conventional imaging (92% vs. 65%, *p* < 0.001) [1]. Next to PSMA-PET imaging, mpMRI of the prostate emerged as a cornerstone in the diagnostic pathway of PCa. A Cochrane meta-analysis found a pooled sensitivity of 0.91 (95% CI: 0.83–0.95) for the detection of PCa ≥ ISUP 2 and a pooled sensitivity of 0.95 (95% CI: 0.87–0.99) for the detection of PCa ≥ ISUP 3 [13]. Furthermore, other strategies such as biomarker testing could potentially complement non-invasive diagnostics. After all, PCa shows some of the highest amounts of cell-free DNA and circulating tumor cells of all solid tumors [14]. However, with these recent advances in clinical risk stratification of PCa, we aimed to investigate whether in cases of very high suspicion of PCa biopsy can be omitted. This taking into consideration that prior biopsy could potentially represent unnecessary delay or potential morbidity and anxiety [15]. In the first observational prospective study addressing this issue, we showed that performing radical prostatectomy on patients with a suspicious DRE, aPSA value ≥10 ng/mL, high suspicion of PCa on mpMRI (PI-RADS 4 or 5), and high suspicion of PCa on PSMA-PET imaging (CT or MRI) through local uptake of PSMA-ligand led to the diagnosis of clinically significant PCa in all patients. There has been no comparable study on this issue until today. Meissner et al. recently described a retrospective case series of 25 patients undergoing radical prostatectomy without prior biopsy [7]. Similar to our study, all of the patients also displayed at least one suspicious lesion on preoperative mpMRI (PI-RADS ≥ 4) and at least one highly suspicious lesion on preoperative PSMA-PET imaging, assessed by a value ≥ 4 on a five-point Likert scale (PET Score) and a maximum standardized uptake value of PSMA-ligand (SUVmax) of ≥ 4.0 [7]. In contrast to our study, the case series also involved patients with PSA levels below 10 ng/mL. The median PSA level of the included patients was 7.3 ng/mL (IQR 3.9–13.0) and 64% of patients had a suspicious DRE [7]. Similar to our results, all of the patients in the study by Meissner et al. were diagnosed with clinically significant PCa [7]. Interestingly, while application of our inclusion criteria showed extracapsular extension in 61% of our patients, Meissner et al. showed extracapsular extension in 40% of their patients [7]. This large number of patients with extracapsular extension could potentially be associated with the high ratio of positive surgical margins in our study (Group 1: 40.7%; Group 2: 50%) compared to Meissner et al. (20%) [7]. It cannot be ruled out that the combination of preoperative histopathology and imaging would have improved surgery planning in terms of nerve-sparing surgery or wide excision surgery. We also presented a cohort of patients undergoing radical prostatectomy without prior biopsy not meeting the inclusion criteria of Group 1 or 2 (Group 3). Patients of Group 3 underwent radical prostatectomy on explicit request and after thorough discussion of possible risks but did not necessarily show elevated PSA levels ≥10 ng/mL, suspicious mpMRI (PI-RADS 4 or 5), or high suspicion of PCa on PSMA-PET imaging (CT or MRI). Similar to Meissner et al., the median PSA of Group 3 was 7.06 ng/mL (IQR 5.9–8.5). Comparing Group 3 to our prospective study Group 1 and Group 2, we also observed a significantly higher ratio of extracapsular extension compared to the retrospective control Group 3 (pT3a or pT3b: 60.6% vs. 11.8%, *p* = 0.005). Furthermore, PCa ISUP grade > 2 was found in a significantly higher ratio of patients of Group 1 and 2 compared to Group 3 (ISUP grade > 2: 66.7% vs. 23.5%, *p* = 0.027). In the retrospective case series by Meissner et al., 68% of patients were diagnosed with PCa ISUP grade > 2 [7].

It should be highlighted that our study has some important limitations that can primarily be attributed to its single-center design and individual patient concept. Avoidance of or advice against prostate biopsy is not the current standard of care in our department. Thus, only a minority of patients treated at our center, who explicitly wished to avoid prostate biopsy, underwent radical prostatectomy without prior biopsy leading to selection bias. Based on the previous notion, it should be acknowledged that we included a relatively small number of patients without equal distribution in the three groups. Importantly, data on functional outcomes, as well as on long-term follow-up, could not be provided. Moreover, high suspicion of PCa on preoperative PSMA-PET imaging was not always objectified based on the SUVmax. However, as we included patients who underwent preoperative imaging not only in our own facility but also in external radiology institutions, we provide real world data on that matter.

## 5. Conclusions

Our findings indicate that radical prostatectomy without prior biopsy is feasible in terms of the diagnosis of clinically significant PCa when proper preoperative risk stratification including mpMRI and PSMA-PET imaging is applied. It is crucial to emphasize that the aim of this study was not to question the importance of prior biopsy in general, but rather support evidence of omitting prior biopsy in clinically clear cases. However, our inclusion criteria led to the diagnosis of a large amount of locally advanced and aggressive PCa with a high likelihood of recurrence and salvage therapy. Certainly, additional studies are warranted to further improve the optimal clinical and imaging criteria for upfront curative treatment of patients without the need for prior biopsy.

## Figures and Tables

**Table 1:** Demographic and clinical patient characteristics. Continuous values are presented as median and IQR; categorical values are given as number (n; %). IQR: inter quartile range; PSA: prostate-specific antigen; PSAd: prostate-specific antigen density; P-Volume: prostate volume; mpMRI: multiparametric magnetic resonance imaging; PI-RADS v2: Prostate Imaging Reporting and Data System Version 2; PSMA PET-CT: prostate-specific membrane antigen positron emission tomography–computer tomography; ISUP: International Society of Urological Pathology. * Comparison between Group 1 + Group 2 versus Group 3.

Characteristics	GROUP 1 n = 27	GROUP 2 n = 6	GROUP 3 n = 17	*p*-Value *
Age [y]	73 (68.5–76.5)	73 (67.3–79.5)	72 (66–76)	*p* = 0.78
PSA [ng/mL]	19.3 (13.9–38.9)	22.25 (16.6–33.1)	7.06 (5.9–8.5)	***p* < 0.001**
PSAd [ng/mL/cm^3^]	0.48 (0.3–0.6)	0.31 (0.2–0.5)	0.12 (0.11–0.14)	***p* < 0.001**
P-Volume [cm^3^]	40.5 (34–67.3)	85 (61–120.3)	37 (32–49)	*p* = 0.25
Positive preoperative mpMRI	27 (100%)	6 (100%)	15 (88.2%)	***p* = 0.04**
PI-RADS 3	0 (0%)	0 (0%)	1 (7%)	
PI-RADS 4	6 (22.2%)	2 (33.3%)	6 (40%)	
PI-RADS 5	21 (77.8%)	4 (66.7%)	8 (53.3%)	
Positive preoperative PSMA PET-CT or -MRI	27 (100%)	0 (0%)	15 (88%)	*p* = 0.56
cN1 on pre-operative imaging	17 (63%)	4 (66.7%)	1 (7%)	***p* < 0.001**
cN1 on PSMA-PET imaging	13 (48.1%)	0 (0%)	1 (7%)	
cN1 on mpMRI	9 (33.3%)	4 (66.7%)	0 (0%)	
cN1 on PSMA-PET and mpMRI	5 (18.5%)	0 (0%)	0 (0%)	
Tumor stage				***p* = 0.005**
pT2a	1 (3.7%)	1 (16.7%)	3 (17.6%)	
pT2c	9 (33.3%)	2 (33.3%)	12 (70.6%)	
pT3a	9 (33.3%)	0 (0%)	0 (0%)	
pT3b	8 (29.6%)	3 (50%)	2 (11.8%)	
ISUP Grade				***p* = 0.027**
1	0 (0%)	0 (0%)	4 (23.5%)	
2	7 (26%)	3 (50%)	9 (52.9%)	
3	10 (37%)	1 (16.7%)	3 (17.6%)	
4	5 (18.5%)	0 (0%)	0 (0%)	
5	5 (18.5%)	2 (33.3%)	1 (5.9%)	
pN1	7/17 (41.2%)	1/4 (25%)	0/1 (0%)	*p* = 0.44
Positive surgical margin	11 (40.7%)	3 (50%)	2 (11.8%)	*p* = 0.061
Tumor infiltration [%]	30 (20–40)	35 (20–57.5)	15 (13.8–20)	***p* = 0.001**
Operative time [min]	77 (71–89)	76.5 (72.3–79.3)	66 (63–72)	***p* < 0.001**
Blood loss [mL]	300 (200–425)	450 (362.5–500)	200 (200–300)	*p* = 0.15
Postoperative hemoglobin Difference [g/dL]	2.4 (2–3.3)	2.1 (1.3–2.7)	1.6 (1.3–2.7)	***p* = 0.03**
Catheterization [d]	9 (7–12.5)	9.5 (7.5–10)	7 (7–8)	*p* = 0.17
Hospital stay [d]	10 (9–13.5)	11.5 (10.3–12)	9 (8–10)	*p* = 0.09

## Data Availability

Research data can be obtained from the corresponding author according to local data privacy laws.
